# Therapeutic Potential of Dental Pulp Stem Cells and Leukocyte- and Platelet-Rich Fibrin for Osteoarthritis

**DOI:** 10.3390/cells9040980

**Published:** 2020-04-15

**Authors:** Melissa Lo Monaco, Pascal Gervois, Joel Beaumont, Peter Clegg, Annelies Bronckaers, Jean-Michel Vandeweerd, Ivo Lambrichts

**Affiliations:** 1 Cardio & Organ Systems (COST), Biomedical Research Institute (BIOMED), Hasselt University, 3590 Diepenbeek, Belgium; pascal.gervois@uhasselt.be (P.G.); jej.beaumont@maastrichtuniversity.nl (J.B.); annelies.bronckaers@uhasselt.be (A.B.); ivo.lambrichts@uhasselt.be (I.L.); 2 Department of Veterinary Medicine, Integrated Veterinary Research Unit (IVRU) - Namur Research Institute for Life Science (NARILIS), University of Namur, 5000 Namur, Belgium; jean-michel.vandeweerd@unamur.be; 3 Maastricht Radiation Oncology (MaastRO) Lab, GROW—School for Oncology and Developmental Biology, Maastricht University, 6229ER Maastricht, The Netherlands; 4 Department of Musculoskeletal and Ageing Sciences, Institute of Lifecourse and Medical Sciences, University of Liverpool, L7 8TX Liverpool, UK; P.D.Clegg@liverpool.ac.uk

**Keywords:** dental pulp stem cells, leukocyte- and platelet-rich fibrin, osteoarthritis, cartilage regeneration, immunomodulation

## Abstract

Osteoarthritis (OA) is a degenerative and inflammatory joint disorder with cartilage loss. Dental pulp stem cells (DPSCs) can undergo chondrogenic differentiation and secrete growth factors associated with tissue repair and immunomodulation. Leukocyte- and platelet-rich fibrin (L-PRF) emerges in regenerative medicine because of its growth factor content and fibrin matrix. This study evaluates the therapeutic application of DPSCs and L-PRF in OA via immunomodulation and cartilage regeneration. Chondrogenic differentiation of DPSCs, with or without L-PRF exudate (ex) and conditioned medium (CM), and of bone marrow-mesenchymal stem cells was compared. These cells showed differential chondrogenesis. L-PRF was unable to increase cartilage-associated components. Immature murine articular chondrocytes (iMACs) were cultured with L-PRF ex, L-PRF CM, or DPSC CM. L-PRF CM had pro-survival and proliferative effects on unstimulated and cytokine-stimulated iMACs. L-PRF CM stimulated the release of IL-6 and PGE2, and increased *MMP-13*, *TIMP-1* and *IL-6* mRNA levels in cytokine-stimulated iMACs. DPSC CM increased the survival and proliferation of unstimulated iMACs. In cytokine-stimulated iMACs, DPSC CM increased *TIMP-1* gene expression, whereas it inhibited nitrite release in 3D culture. We showed promising effects of DPSCs in an in vitro OA model, as they undergo chondrogenesis in vitro, stimulate the survival of chondrocytes and have immunomodulatory effects.

## 1. Introduction

Articular cartilage (AC) plays key roles in the function of diarthrodial (synovial) joints [1,2]. Cartilage injuries are very common, predominantly in young and active athletes, and particularly in the knee joint [3,4,5]. They are often considered as risk factors for the development of osteoarthritis (OA) in later life, a degenerative and inflammatory condition of the synovial joint with irreversible cartilage loss [2]. OA results in disability, particularly in elderly people and is associated with a large socio-economic burden [6,7]. OA is more prevalent in the female population and increases with age [7]. In people over 60 years of age, it is estimated that 9.6% of men and 18% of women have symptomatic OA [8]. Unfortunately, long-lasting regeneration of damaged AC remains an unmet clinical need. Current treatment strategies aim to relieve pain and clinical signs associated with inflammation. However, patients show no long-term improvements [9]. With the aim to restore the damaged cartilage tissue, matrix-induced autologous chondrocyte implantation (MACI), a Food and Drug Administration (FDA)-approved technique, has been developed [10]. However, there are several disadvantages such as iatrogenic damage and high costs [11,12,13]. To overcome these problems, the use of innovative autologous biological tissue engineering techniques using stem cells forms an area of large interest in an attempt to achieve AC regeneration. 

Previous preclinical studies focused on the use of induced pluripotent stem cells (iPSCs) and mesenchymal stem cells (MSCs) to repair AC, demonstrating beneficial effects mediated via different mechanisms (as previously reviewed by our group [2]). However, because of the ethical implications regarding the usage of iPSCs and the invasive nature of bone marrow-derived MSC (BM-MSC) isolation, an alternative cell source is of particular interest: dental pulp stem cells (DPSCs) originating from the neural crest-derived mesenchyme residing in the dental pulp [14,15]. Since they are isolated from extracted human third molars, DPSCs can be obtained with minimal donor site morbidity and iatrogenic damage. DPSCs have been classified as MSCs based upon the International Society for Cellular Therapy (ISCT) criteria [16]. Apart from the opportunity of DPSCs to provide a cell replacement treatment, they show therapeutic potential in OA through paracrine and trophic influences on endogenous cells. Current evidence indicates that DPSCs can be differentiated into cartilage-producing cells [17] and secrete numerous growth factors associated with tissue repair and immunomodulation, including vascular endothelial growth factor (VEGF), monocyte chemoattractant protein (MCP)-1, transforming growth factor-beta (TGF-β) and tissue inhibitors of metalloproteinases (TIMPs) [18,19,20]. In addition, their immunomodulatory capacity makes them strong contenders to be used in inflammatory disorders [21], such as OA. Interestingly, intra-articular injection of DPSCs resulted in anti-inflammatory effects in rheumatoid arthritis (RA) [22]. Co-culture of costal chondrocytes and DPSCs combined with fibroblast growth factors (FGF)-9 showed enhanced chondrogenesis and reduced ossification in tissue-engineered cartilage [23]. However, until now, no chondrocyte-salvaging or -stimulating properties have been attributed to DPSCs. 

In addition, different growth factors including TGF-β, basic (b) FGF, VEGF, bone morphogenetic proteins (BMPs) and platelet derived growth factor (PDGF) have been described to have a beneficial effect on hyaline cartilage repair [24]. Platelets are a natural reservoir of such growth factors within the human body [25]. Platelet concentrates such as platelet-rich plasma (PRP) and leukocyte- and platelet-rich fibrin (L-PRF), are known to produce a plethora of autologous growth factors and cytokines [26]. In recent years, first generation platelet-rich biomaterials such as autologous PRP have been widely studied in order to realise AC repair [27]. In vitro studies demonstrated their positive effects on chondrocyte proliferation and deposition of cartilage matrix [28,29]. Several preclinical animal studies revealed positive effects on cartilage repair induced by PRP [27]. In contrast to PRP, L-PRF is a second generation platelet concentrate which can be produced rapidly by the collection of autologous blood after one single centrifugation step and without anticoagulants [26,30]. The generated product is a fibrin clot consisting of three components; leukocytes, platelets and a supportive fibrin matrix [31]. Leukocytes and platelets progressively release a high concentration of cytokines and growth factors respectively over time [31,32]. L-PRF might be applied in cartilage engineering studies because of its supportive fibrin matrix, while the leukocytes present in L-PRF might be important in immunomodulatory mechanisms via cytokine secretion. To date, numerous studies have mainly investigated the cartilage regenerative potential of other platelet derivatives, such as PRP [33]. In vitro studies of the chondrogenic potential of L-PRF are limited [34]. Some trophic and protective effects by PRF on chondrocytes have previously been demonstrated [35,36,37], and one in vitro study showed the chondro-inductive effect of the eluate from fibrin-rich plasma membrane on a stem cell population [38].

Because of the chondrogenic differentiation potential and immunomodulatory properties of DPSCs, and the growth factor-rich content of L-PRF, we hypothesise that DPSCs and L-PRF can both enhance cartilage regeneration in vitro and have immunomodulatory effects. In the current study, first, we investigated the in vitro chondrogenic differentiation potential of human DPSCs compared to human BM-MSCs. Second, we evaluated whether L-PRF stimulated the chondro-induction of both MSC types in a three-dimensional (3D) cell-culture system. Third, we assessed the effect of growth factor release of DPSCs and L-PRF on healthy chondrocytes and tumour necrosis factor-alpha (TNF-α) and interleukin-1beta (IL-1β)-stimulated chondrocytes in vitro, on viability, OA-related gene expression, cartilage-specific extracellular matrix (ECM) deposition and inflammatory cytokine secretion. 

## 2. Materials and Methods

### 2.1. Human Stem Cell Isolation and Culture

Human third molars were obtained with written informed consent from patients (*n* = 16) of both genders (15–20 years of age) undergoing an extraction procedure for orthodontic reasons at Ziekenhuis Oost-Limburg (ZOL, Genk, Belgium). Written informed consent of minor patients was acquired via their custodians. The study was conducted in accordance with the Declaration of Helsinki, and the study protocol was approved by the medical ethical committee of Hasselt University (Belgium, protocol 13/0104U, date of approval 3 February 2014). The pulp tissue was obtained by means of forceps after mechanically fracturing the teeth. Next, the pulp tissues were minced into small pieces (1–2 mm^3^) and DPSCs were isolated via the explant method [17]. Cells were maintained in minimal essential medium, alpha modification (αMEM, Sigma-Aldrich, St. Louis, MO, USA) supplemented with 100 U/mL Penicillin and 100 μg/mL Streptomycin (Sigma-Aldrich), 2 mM l-glutamine (Sigma-Aldrich) containing 10% heat-inactivated foetal bovine serum (FBS) (Biowest, Nuaillé, France). 

BM-MSCs of three different donors (both male and female), between 6 and 12 years old, were kindly provided by Prof. Dr. Cathérine Verfaillie (Stem Cell Institute, KU Leuven, Leuven, Belgium). BM-MSCs were kept in high-glucose Dulbecco’s modified Eagle’s medium (DMEM, Sigma-Aldrich) supplemented with 100 U/mL Penicillin and 100 μg/mL Streptomycin containing 10% heat-inactivated FBS.

All stem cells were routinely screened in our lab for the expression of the following markers: CD34 (negative), CD44, CD45 (negative), CD90, CD105 and Stro-1 (negative) [17]. All cell cultures were maintained at 37 °C in a humidified atmosphere containing 5% CO_2_. The culture medium was changed every 2–3 days and all cultures were regularly monitored with an inverted phase-contrast microscope Nikon Eclipse TS100 (Nikon Co., Shinjuku, Tokyo, Japan) equipped with a Jenoptik ProgRes C3 camera (Jenoptik, Jena, Germany) with corresponding ProgRes Capture Pro 2.7 software. When reaching 80–90% confluence, cells were harvested using 0.05% trypsin/EDTA (Sigma-Aldrich) and sub-cultured for further experiments. All experiments were conducted with DPSCs between passages 2 and 8.

### 2.2. Isolation and Culture of Immature Murine Articular Chondrocytes

Immature murine articular chondrocytes (iMACs) were isolated based upon a previously published protocol by Gosset et al. [39] and according to the animal welfare guidelines of the ethical committee of Hasselt University (ID 201762K, date of approval 11 November 2017). In short, after euthanasia of 5–6-day-old C57BL/6 wild type mice (*n* = 219), femoral heads, femoral condyles and tibial plateaus were isolated from the hind limbs and placed in phosphate buffered saline (PBS, Lonza, Basel, Switzerland). Isolated cartilage pieces were then incubated twice in 3 mg/mL collagenase D (Sigma-Aldrich) in low glucose DMEM (Sigma-Aldrich) supplemented with 50 U/mL Penicillin, 50 μg/mL Streptomycin and 2 mM l-glutamine for 45 min at 37 °C in 5% CO_2_. Cartilage pieces were then incubated 0.5 mg/mL collagenase D in standard culture medium overnight at 37 °C in 5% CO_2_. Afterwards, cartilage fragments were passed through 25 mL, 10 mL, 5 mL and 2 mL pipettes to disperse any cell aggregates. After passing through a 70-μm cell strainer, the cells were centrifuged at 400× *g* for 10 min. Cells were resuspended in iMAC standard culture medium supplemented with 10 % heat-inactivated FBS. 

Phenotypic characterization was performed by means of immunocytochemistry (ICC) and histological staining. In short, 26.32 × 10^3^ cells/cm^2^ were seeded on glass or plastic (Thermanox^®^; Electron Microscopy Sciences, Hatfield, PA, USA) cover slips for 96 h in standard culture medium supplemented with 10% FBS. Afterwards, they were fixed using 4% paraformaldehyde (PFA) for 20 min for ICC or using 2% glutaraldehyde in 0.05 M cacodylate buffer (pH 7.3) at 4 °C for transmission electron microscopy (TEM) processing. Immune-reactivity for collagen type II was demonstrated by ICC. Culture purity was assessed by determining the fraction of collagen type 2-positive cells using ImageJ software (The National Institute of Health, MD, USA). The presence of proteoglycans (PGs) was demonstrated via alcian blue, toluidine blue and safranin O staining. All experiments were performed with freshly isolated iMACs.

### 2.3. L-PRF Isolation

Blood samples were obtained from 11 healthy donors from both genders (aged 23–37) (*n* = 11) with written informed consent. The study protocol and consent procedure were approved by the medical ethical committee from Hasselt University and the Clinical Trial Centre from KU Leuven (S58789/B322201628215, date of approval 21 March 2016). All experiments were performed in accordance with relevant guidelines and regulations. Blood samples were drawn by venipuncture and collected in glass-coated plastic tubes (VACUETTE 9 mL Z Serum Clot Activator Tubes, Greiner Bio-One, Vilvoorde, Belgium). Samples were immediately centrifuged for 12 min at 2700 rpm (400× *g*) (IntraSpin^™^ Centrifuge, Intra-Lock, Boca Raton, FL, USA). The L-PRF clots were removed from the tubes using sterile forceps and separated from the red blood cell phase with an iris spatula (Fine Science Tools, Heidelberg, Germany). 

### 2.4. L-PRF Conditioned Medium and Exudate

For the production of L-PRF conditioned medium (L-PRF CM), L-PRF clots were placed in 6 mL of serum-free low glucose DMEM or DMEM/F12 (Thermo Fisher Scientific, Erembodegem, Belgium) supplemented with 2 mM L-glutamine, 50 or 100 U/mL Penicillin and 50 or 100 μg/mL Streptomycin. After 96 h, the medium was collected, centrifuged for 6 min at 300× *g*, sterile filtered (0.2 µm, Sarstedt, Nümbrecht, Germany) and stored at −80 °C until further use. For L-PRF exudate (L-PRF ex) collection, the L-PRF clots were brought to a sterile box (Xpression™ Fabrication Box, Intra-Lock) and compressed, thereby releasing the exudate, which was collected, sterile filtered and stored at −80 °C until further usage.

### 2.5. Chondrogenic Differentiation

Chondrogenic differentiation of DPSCs and BM-MSCs was induced according to the manufacturer’s instructions (StemXVivo Human/Mouse Chondrogenic Supplement, R&D systems, BioTechne, Minneapolis, MN, USA). A pellet containing 2.5 × 10^5^ cells in a 15 mL conical tube was subjected to chondrogenic differentiation medium consisting of DMEM/F12 supplemented with 1% insulin transferrin selenite (R&D systems) and 1% chondrogenic supplement (R&D systems). This supplement consists of dexamethasone, ascorbate-phosphate, proline, pyruvate and TGF-β3 with concentrations determined and validated by the manufacturer. To determine the effect of L-PRF on the chondrogenic differentiation, L-PRF ex (3%) and L-PRF CM (5% and 25%) were added to the differentiation medium. Positive and negative controls contained standard differentiation medium with or without the chondrogenic supplement respectively. Every 2–3 days, the medium was changed. The cells were allowed to differentiate for 21 days, after which the pellets were either fixed with 4% PFA for immunohistochemical (IHC) analysis or with 2% glutaraldehyde in 0.05 M cacodylate buffer (pH 7.3) at 4 °C for TEM processing. Percentage alcian blue and aggrecan stained area was quantified using Image J (The National Institute of Health, MD, USA). 

### 2.6. DPSC Conditioned Medium

Conditioned medium of DPSCs (DPSC CM) was prepared by seeding human DPSCs at a density of 20 × 10^3^ cells/cm^2^ in standard culture medium supplemented with 10% FBS. Cells were allowed to attach overnight. Afterwards, cells were rinsed twice with PBS and 1 mL/5 cm^2^ iMAC serum-free standard culturing medium was added. 48 h later, the medium was collected, centrifuged at 161× *g* for 6 min and stored at −80 °C.

### 2.7. Cell Survival and Proliferation Assay

iMACs were seeded in triplicate in flat bottom 96 well plates at a density of 19.69 × 10^3^; cells/cm^2^ or 29.41 × 10^3^ cells/cm^2^ for survival and proliferation assays respectively and were allowed to attach overnight. Hereafter, cells were washed twice with PBS and culture medium supplemented with L-PRF ex (1%, 3%, 5%), L-PRF CM (5%, 25%, 50%), or DPSC CM was added. For survival assays, the cells were cultured in serum-free conditions. For proliferation assays, experimental conditions were supplemented with 2% FBS. Negative and positive controls consisted of iMACs cultured in serum-deprived medium (0% or 2% for survival and proliferation respectively) or medium supplemented with 10% FBS respectively. 

For cytokine-stimulated iMACs, cells were seeded at a density of 29.41 × 10^3^ cells/cm^2^ and were allowed to adhere overnight. Subsequently, cells were washed twice with PBS and stimulated with the inflammatory cytokines recombinant mouse TNF-α (10 ng/mL) and recombinant mouse IL-1β (10 ng/mL) (Immunotools, Friesoythe, Germany) for 24 h. Hereafter, experimental conditions were added, including inflammatory cytokines TNF-α and IL-1β (10 ng/mL). Unstimulated conditions received no cytokines. 

The effect of L-PRF ex, L-PRF CM and DPSC CM on iMAC viability was evaluated using propidium iodide (PI, Sigma-Aldrich). After 24, 48, or 72 h, cells were lysed using Reagent A100 (Chemometec, Lillerød, Denmark). Next, cells were incubated with PI (diluted 1/50 in Reagent B (Chemometec)) for 15 min in the dark. Solutions were transferred to a black 96-well plate with clear bottom (Greiner bio-one) and fluorescence intensity was measured at an excitation wavelength of 540 nm and an emission wavelength of 612 nm (FLUOstar OPTIMA, BMG Labtech, Ortenberg, Germany).

### 2.8. Reverse Transcriptase Quantitative Polymerase Chain Reaction

iMACs were seeded at a cell density of 52.63 × 10^3^ cells/cm^2^ and left to adhere overnight. The cells were subsequently washed twice with PBS and stimulated with inflammatory cytokines recombinant mouse TNF-α (10 ng/mL) and recombinant mouse IL-1β (10 ng/mL) for 24 h. Afterwards, experimental conditions were added containing TNF-α and IL-1β (10 ng/mL). Unstimulated conditions received no cytokines. All cells were cultured in 2% FBS. 

After 24 h, medium was collected, centrifuged and stored at −80 °C for enzyme-linked immunosorbent assay (ELISA) experiments, while RNA was extracted from total cell lysates using the RNeasy Mini Kit (74104, Qiagen, Venlo, the Netherlands) according to the manufacturer’s instructions. After reverse transcription to cDNA using qScript cDNA Supermix (Quanta Bioscience, Carlsbad, CA, USA), a quantitative PCR was conducted on a StepOnePlus detection system (Applied Biosystems, Foster City, CA, USA) using standardised cycling conditions (20 s at 95 °C, 40 cycles of 3 s at 95 °C and 30 s at 60 °C). Primer sequences are listed in Supplementary Table S1.

### 2.9. Enzyme-Linked Immunosorbent Assay

ELISAs were performed for IL-6 and prostaglandin E2 (PGE2) (R&D systems). ELISAs were performed according to the guidelines of the manufacturer. The absorbance of the end product was measured with a plate reader (FLUOstar OPTIMA and iMARK Microplate Reader, Biorad, Temse, Belgium). To ensure that the measured concentrations in L-PRF ex, L-PRF CM and DPSC CM were iMAC-derived, the conditions were included in the ELISA experiment as a control.

### 2.10. Nitrite Measurements

iMACs were seeded at a density of 52.63 × 10^3^ cells/cm^2^ and were allowed to adhere for 24 h. Cells were subsequently washed twice met PBS and stimulated with the inflammatory cytokines TNF-α (10 ng/mL) and IL-1β (10 ng/mL). After 24 h, experimental conditions were added containing TNF-α and IL-1β (10 ng/mL). Unstimulated conditions received no cytokines. All cells were cultured in 2% FBS. After another 24 h, the medium was collected, centrifuged and stored at −80 °C. 

Nitrite was quantified using the Griess Reagent System (Promega Benelux B.V., Leiden, The Netherlands) according to the manufacturer’s guidelines. Absorbance was measured at a wavelength of 540 nm using a plate reader (FLUOstar OPTIMA).

### 2.11. Three-Dimensional Culture of iMACs

For cytokine-stimulated iMAC pellets, 5 × 10^5^ iMACs were washed twice and resuspended in culture medium containing recombinant mouse TNF-α (10 ng/mL) and recombinant mouse IL-1β (10 ng/mL). Cells were centrifuged in 15 mL polypropylene tubes at 400× *g* and maintained at 37 °C under 5% CO_2_. The caps of the tubes were loosened to allow for air exchange. 24 h later, the medium was replaced for the experimental conditions with TNF-α and IL-1β (10 ng/mL). Unstimulated conditions received no cytokines. All conditions were cultured in 2% FBS. 72 h later, the medium was collected, centrifuged and stored at −80 °C for nitrite measurements, while pellets were fixed with 4% PFA for IHC. 

### 2.12. Immunocytochemical Staining

For collagen type II expression in iMACs, permeabilisation and blocking occurred simultaneously with 10% protein block (DAKO, Glostrup, Denmark) and 0.2% Triton in PBS for one hour. Cells were then incubated with an anti-collagen type II antibody (ab34712, polyclonal, 1:100, Abcam, Cambridge, UK) diluted in 10% protein block in PBS for one hour at room temperature (RT). Afterwards, they were incubated with the Alexa 555-labelled donkey anti-rabbit IgG (A31572, Thermo Fisher Scientific) diluted 1/500 in PBS for 30 min. Nuclei were stained with 4′,6-diamidino-2-phenylindole (DAPI, Thermo Fisher Scientific) for 10 min. Samples were mounted using fluorescence mounting medium (DAKO). Pictures were taken with a Leica DM4000 B Microscope (Leica Microsystems, Wetzlar, Germany).

### 2.13. (Immuno)histology

#### 2.13.1. Immunohistochemistry

Cartilage pellets were embedded in paraffin and 7 μm thick sections were cut. Samples were deparaffinised in xylene and ethanol baths (xylene: 2 times 5 min, ethanol: 100%, 100%, 95%, 80%, 70%, 50%, 2 min each). Antigen retrieval was performed by heating the samples three times for 5 min in 1× target retrieval solution (DAKO). In case of 3,3’-Diaminobenzidine (DAB, DAKO) staining, peroxidase block (DAKO) was used for 20 min. Next, nonspecific binding of the antibodies was inhibited with protein block (DAKO) for 30 min at RT. Samples were then incubated with an anti-aggrecan antibody (ab186414, clone number EPR14664, 1:500, Abcam) diluted in 10% protein block in PBS for one hour at RT. Subsequently, samples were incubated with the advance HRP Link System (K4067, DAKO) for 30 min at RT. Hereafter, samples were incubated with DAB for 5 min and counterstained with haematoxylin for 8 min after which they were washed with running tap water for 20 min.

#### 2.13.2. Histology

For histological analyses routinely used safranin O, alcian blue, toluidine blue and Masson’s trichrome staining were performed (See Supplemental Materials and Methods). 

All samples were dehydrated in ethanol and xylene (ethanol: 70%, 80%, 95%, 100%, 100%, 100% xylene, 100% xylene, 2 min each) and mounted using DPX (Merck, Darmstadt, Germany). Slides were visualised with the Mirax slide scanner (Carl Zeiss NV-SA, Zaventem, Belgium) using the Mirax scan software. Photos of scanned slides were made with the Mirax viewer (Carl Zeiss NV-SA) or images were taken with a Leica DM2000 LED Microscope. 

### 2.14. Transmission Electron Microscopy 

Samples were processed for TEM imaging as described previously [40]. After fixation, the fixative was aspirated with a glass pipette, and samples were postfixed in 2% osmium tetroxide for one hour. Subsequently, samples were placed through a dehydrating series of graded concentrations of acetone. Dehydrated samples were impregnated overnight in a 1:1 mixture of acetone and araldite epoxy resin at RT. After impregnation, samples were embedded in araldite epoxy resin at 60 °C and monolayer samples were embedded in araldite according to the popoff method [41]. Ultrathin sections (0.06 μm) were mounted on 0.7% formvar-coated copper grids (Aurion, Wageningen, the Netherlands), contrasted with 0.5% uranyl acetate and a stabilised solution of lead citrate using a Leica EM AC20 (Leica). Samples were observed using a Philips EM 208 transmission electron microscope (Philips, Eindhoven, The Netherlands) equipped with a Morada Soft Imaging System camera with corresponding iTEM-FEI software (Olympus SIS, Münster, Germany). 

### 2.15. Statistical Analysis

Statistical analysis was performed using Graphpad Prism 7.04 software (Graphpad, San Diego, CA, USA). Normality was tested using the Shapiro-Wilk and the D’Agostino and Pearson normality test. Normal distributed data were tested with one-way analysis of variance (ANOVA) or two-way ANOVA and Dunnet’s multiple comparison post-test. Nonparametric data were analysed with the Kruskal-Wallis test followed by Dunn’s post-test. Any *p*-value ≤ 0.05 was considered to be statistically significant. All data were presented as mean ± standard error of mean (S.E.M.).

## 3. Results

### 3.1. Differences in Chondrogenic Differentiation Potential Between BM-MSCs and DPSCs and the Effect of Exposure to L-PRF During Chondrogenesis

**Figure 1:**
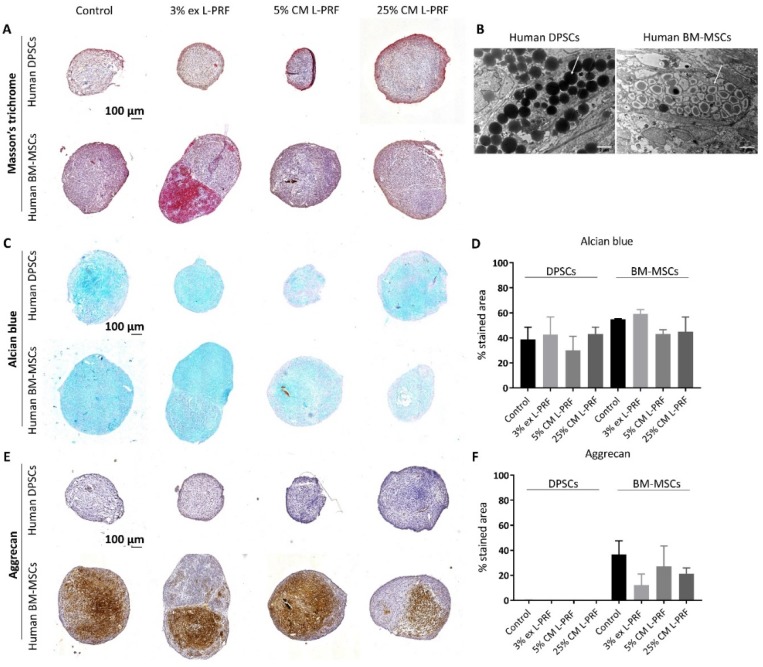
Differences in chondrogenic differentiation potential between human dental pulp stem cells (DPSCs) and human bone marrow-derived MSCs (BM-MSCs) and the effect of exposure to L-PRF during chondrogenesis. After 21 days of exposure to L-PRF ex (3%) or L-PRF conditioned medium (CM) (5% and 25%), cartilage-specific protein expression in differentiated pellets was evaluated using (immuno)histological staining (*n* = 3). (**A**) Masson’s trichrome staining revealed the presence of abundant extracellular matrix (ECM) in micropellets derived from both differentiated stem cell types. (**B**) Ultrastructural analyses of the micropellets of both cell types showed the presence of dense matrix-filled vesicles (arrowheads). (**C**) Glycosaminoglycan (GAG) production was assessed by means of alcian blue staining. (**D**) L-PRF ex or L-PRF CM stimulation did not enhance the GAG area percentage. (**E**) Immunohistochemical (IHC) revealed that aggrecan expression was present in differentiated BM-MSCs, but absent in the DPSC-derived pellets. (**F**) Aggrecan area percentage was not enhanced by L-PRF ex or L-PRF CM exposure. Scale bars A, C, E = 100 μm; B = 2 μm. Data in D and F are represented as mean ± S.E.M.

In order to compare the chondrogenic differentiation potential between human DPSCs and BM-MSCs, cells were subjected to a 3D chondrogenic differentiation system over 21 days. To test the effect of exposure to L-PRF during chondrogenic differentiation, cells were subjected to the same 3D differentiation system, but supplemented with L-PRF ex (3%) or L-PRF CM (5% and 25%) for 21 days (*n* = 3). Following the three week culture, both cell types formed compact 3D micromasses under all experimental conditions (Figure 1A). IHC revealed abundantly present ECM surrounding both differentiated stem cell types (Figure 1A). Ultrastructural analyses of the 3D micropellets of both cell types showed the presence of dense matrix-filled vesicles, suggesting glycosaminoglycan (GAG) production (Figure 1B, arrowheads). This was supported by the alcian blue staining which demonstrated the presence of GAGs in the ECM of both differentiated MSC types (Figure 1C). Quantitative analysis of GAG production demonstrated no significant difference between DPSCs and BM-MSCs after 21 days of differentiation (Figure 1D). Moreover, when the chondrogenic differentiation medium was supplemented with L-PRF ex or L-PRF CM, the percentage of the alcian blue-stained area in micropellets derived from both cell types was not significantly different (Figure 1D). Aggrecan expression could only be detected in differentiated BM-MSCs and remained absent in DPSC-derived pellets (Figure 1E,F). Exposure to L-PRF ex or CM did not significantly augment the aggrecan expression in cartilage spheres derived from BM-MSCs (Figure 1F). The control pellet resulted in 36.69% ± 10.89% aggrecan-positive stained area, while 5% L-PRF CM caused 27.24% ± 16.22% aggrecan-positive area compared to 21.34% ± 4.61% aggrecan-stained area in BM-MSCs supplemented with 25% L-PRF CM.

### 3.2. Phenotypical and Ultrastructural Characterization of Immature Murine Articular Chondrocytes

**Figure 2:**
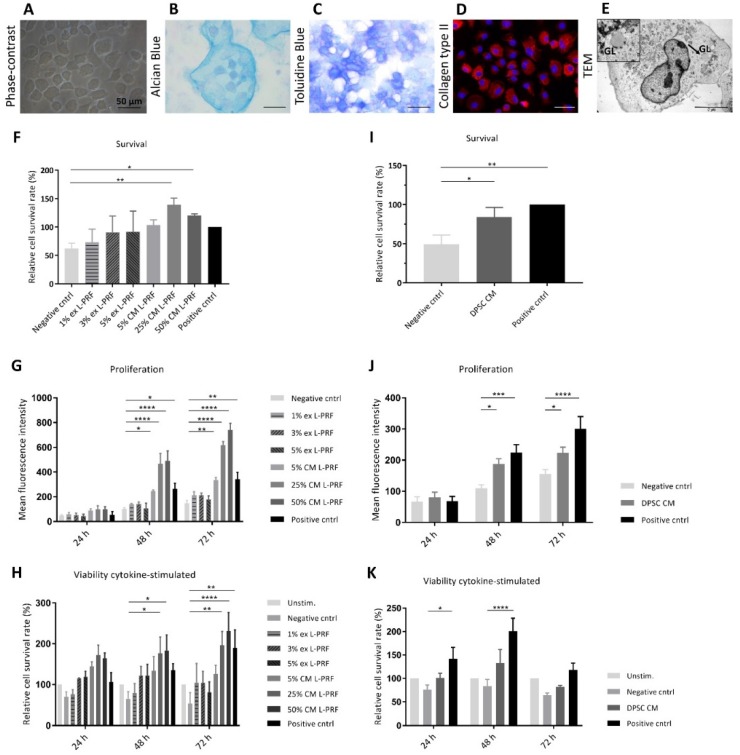
Phenotypic characterization of immature murine articular chondrocytes (iMACs) and the effect of L-PRF ex, L-PRF CM and DPSC CM on iMAC survival, proliferation and chondrocyte viability in TNF-α- and IL-1β-stimulated conditions. (**A**) Phase contrast micrographs of mouse iMACs show a rounded, polygonal morphology. (**B**–**D**) Histological staining revealed the production of sulphated PGs, while immunofluorescence staining demonstrated collagen type II expression. (**E**) Transmission electron microscopy (TEM) showed a rounded, spherical morphology with abundant rough endoplasmic reticulum, mitochondria and glycogen-rich (GL) vacuoles (insert). The effect of the secretome of L-PRF and DPSCs on unstimulated iMAC survival, proliferation and cytokine-stimulated iMAC viability were evaluated by means of a PI assay (**F**–**K**). (**F**) 25% and 50% L-PRF CM had a significant pro-survival effect on iMACs after 24 h compared to the negative control (*n* = 5). (**G**) 5%, 25% and 50% L-PRF CM had a significant proliferative effect on iMACs after 48 h and 72 h compared to the negative control (*n* = 4). (**H**) 25% and 50% L-PRF CM significantly increased the viability of TNF-α- and IL-1β-stimulated iMACs after 48 h and 72 h (*n* = 5 for 24 h, *n* = 6 for 48 h, *n* = 6 for 72 h). (**I**) DPSC CM had a significant pro-survival effect on iMACs after 24 h compared to the negative control (*n* = 8). (**J**) DPSC CM significantly increased the proliferation of iMACs after 48 h and 72 h (*n* = 7 for 24 h, *n* = 8 for 48 h, *n* = 9 for 72 h). (**K**) TNF-α- and IL-1β-stimulated iMAC viability follows an increasing trend after exposure to DPSC CM, although not statistically significant. (*n* = 8 for 24 h, *n* = 10 for 48 h, *n* = 8 for 72 h). Scale bars A, B, C and D = 50 μm. Scale bar E: 5 μm (original magnification: 5,600). Data are represented as mean ± S.E.M. *. *p* ≤ 0.05. **. *p* ≤ 0.01. ***. *p* ≤ 0.001. ****. *p* ≤ 0.0001.

iMACs were isolated from the femoral heads, femoral condyles and tibial plateau from hind limbs of 5–6-day-old wild type C57BL/6 mice. Phase contrast images revealed a rounded and polygonal morphology with a granular cytoplasm (Figure 2A). Expression of the main markers of chondrocyte phenotype was assessed via (immuno)histology. Alcian blue and toluidine blue staining show the presence of PG components (Figure 2B,C), while ICC demonstrated collagen type II expression by iMACs (Figure 2D). The average culture purity was 93.24% ± 1.33% (*n* = 3). Together, iMACs synthesise type II collagen and sulphated PGs in vitro after 4 days, showing the isolation of functional chondrocytes. Ultrastructurally, chondrocytes were characterised by a rounded, spherical morphology with ample rough endoplasmic reticulum, mitochondria and glycogen-rich vacuoles (Figure 2E and insert). 

### 3.3. Effect of Secreted Factors of DPSCs and L-PRF on Healthy Chondrocyte Survival and Proliferation and Viability of TNF-α- and IL-1β-Stimulated iMACs

In order to evaluate the influence of L-PRF ex, L-PRF CM and DPSC CM on the viability of unstimulated or cytokine-stimulated iMACs, a PI test was employed at different time points (Figure 2F–K). After 24 h, serum deprivation decreased survival compared to iMACs cultured in high serum conditions (Figure 2F,I). This effect could not be prevented by supplementation of L-PRF ex to iMACs (Figure 2F). In contrast, the highest L-PRF CM concentrations (25% and 50%) had a significant pro-survival effect compared to the negative control condition and this was demonstrated to have a proliferative influence when serum was absent (139% ± 11.93% for 25% L-PRF CM and 120.2% ± 3.02% for 50% L-PRF CM) (Figure 2F). When 2% serum was supplemented, all L-PRF CM concentrations (5%, 25% and 50%) significantly increased iMAC proliferation at 48 h and 72 h (Figure 2G). When iMACs were stimulated with TNF-α and IL-1β, 25% and 50% L-PRF CM showed a statistically significant increased viability compared to the cytokine-stimulated negative control at 48 h and 72 h (177% ± 39.51% and 183.7% ± 38.24% for 25% and 50% L-PRF CM respectively compared to 65.1% ± 17.4% for the stimulated negative control for 48 h, 196.4% ± 33.86% and 231.2% ± 45.66% for 25% and 50% L-PRF CM respectively compared to 53.41% ± 26.7% for the stimulated negative control at 72 h) (Figure 2H). L-PRF ex did not exert any stimulating effects on proliferation or viability of neither unstimulated nor cytokine-stimulated iMACs. In serum-deficient conditions, DPSC CM significantly stimulated iMAC survival compared to the negative control (84.16% ± 12.06% compared to 49.08% ± 11.81%) after 24 h (Figure 2I). In 2% serum conditions, iMAC underwent a significant increased proliferation compared to the negative control after 48 h and 72 h when cultured in DPSC CM (Figure 2J). When cytokine-stimulation was implemented, iMAC viability followed an increasing trend when cultured in DPSC CM at every time point, although this effect was not significant (Figure 2K).

### 3.4. Effect of Secreted Factors of DPSCs and L-PRF on OA-related mRNA Expression of Unstimulated and TNF-α- and IL-1β-Stimulated iMACs

Expression levels of chondrocyte-markers were investigated in unstimulated iMACs cultured with 3% L-PRF ex, 25% L-PRF CM and DPSC CM after 24 h (Figure S1). *Aggrecan* mRNA levels were significantly decreased upon supplementation of 3% L-PRF ex and 25% L-PRF CM (Figure S1A). 25% L-PRF CM significantly decreased mRNA levels of *collagen type II α 1* (Figure S1B). *TGF-β* mRNA levels were not significantly altered by L-PRF ex, L-PRF CM and DPSC CM (Figure S1C). Matrix metalloproteinase *(MMP)-13* was significantly upregulated in iMACs cultured with 25% L-PRF CM compared to control (Figure S1D), while *TIMP-1* mRNA expression levels were significantly upregulated by the supplementation of 25% L-PRF CM and DPSC CM (Figure S1E). 

**Figure 3:**
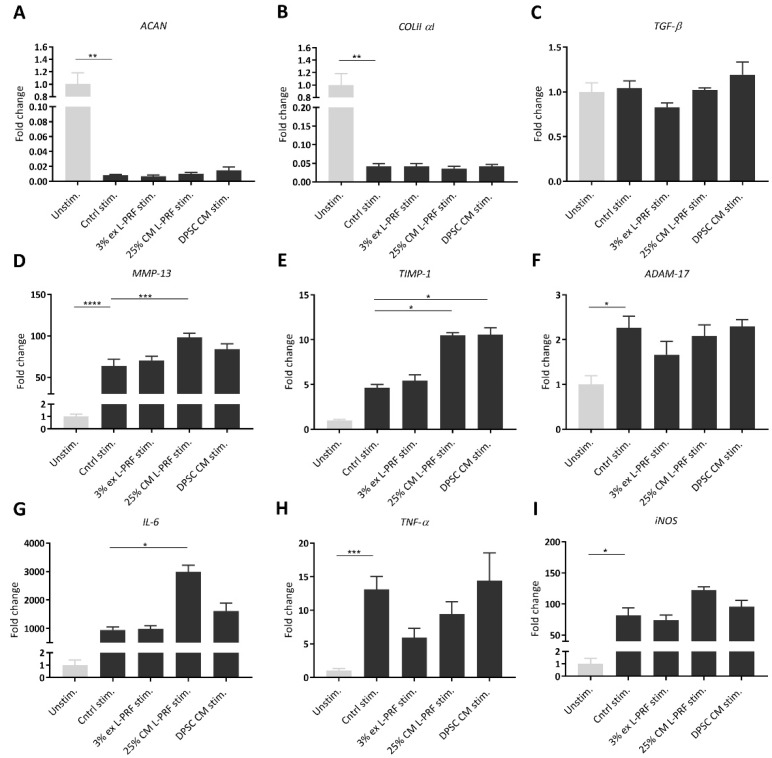
Effect of L-PRF ex, L-PRF CM and DPSC CM on TNF-α- and IL-1β- stimulated iMAC OA-related gene expression. Relative mRNA levels were determined by RT-qPCR of unstimulated and cytokine-stimulated iMACs exposed to 3% L-PRF ex, 25% L-PRF CM and DPSC CM. (**A**,**B**) Chondrocyte marker genes, *aggrecan* and *collagen type II α 1*, were significantly downregulated by cytokine stimulation, but not significantly altered by L-PRF ex, L-PRF CM and DPSC CM. (**C**) *TGF-β* mRNA levels were not altered upon exposure to cytokines, nor in combination with L-PRF ex, L-PRF CM or DPSC CM. (**D**) *MMP-13* was significantly upregulated after cytokine stimulation, while 25% L-PRF CM further increased *MMP-13* mRNA levels compared to the stimulated control. (**E**) *TIMP-1* was upregulated by the supplementation of pro-inflammatory cytokines combined with 25% L-PRF CM and DPSC CM. (**F**) *ADAM-17* expression was significantly upregulated after cytokine stimulation but not altered after exposure to L-PRF ex, L-PRF CM and DPSC CM. (**G**) 25% L-PRF CM significantly augmented the *IL-6* mRNA levels compared to the stimulated control. (**H**,**I**) *TNF-α* and *iNOS* mRNA levels were upregulated upon exposure to cytokines, but not altered by the supplementation of L-PRF ex, L-PRF CM or DPSC CM. Data correspond to *n* = 6 for L-PRF ex and L-PRF CM and *n* = 7 for DPSC CM. Data are represented as mean ± S.E.M. *. *p* ≤ 0.05. **. *p* ≤ 0.01. ***. *p* ≤ 0.001. ****. *p* ≤ 0.0001.

After iMACs were cytokine stimulated for 24 h and cultured in experimental conditions for another 24 h, gene expression levels of OA-related markers *aggrecan*, *collagen type II α 1*, *TGF-β*, *MMP-13*, *TIMP-1*, a disintegrin and metalloproteinase (*ADAM)-17*, *IL-6*, *TNF-α* and inducible nitric oxide synthase (*iNOS*) were measured. As shown in Figure 3, reverse transcriptase quantitative polymerase chain reaction (RT-qPCR) results showed that cytokine stimulation of iMACs significantly decreased cartilage-specific mRNA levels, such as *aggrecan* and *collagen type II α 1*, compared to unstimulated iMACs after 24 h (Figure 3A,B). No significant increase in *aggrecan* or *collagen type II α 1* could be observed when iMACs were cultured with 3% L-PRF ex, 25% L-PRF CM or DPSC CM (Figure 3A,B). mRNA levels of *TGF-β*, a growth factor playing indispensable roles in cartilage integrity and homeostasis, were also measured using RT-qPCR and were not significantly altered (Figure 3C). TNF-α and IL-1β stimulation of iMACs increased mRNA levels of the chondrocyte maturation marker *MMP-13* compared to unstimulated iMACs (Figure 3D). Moreover, 25% L-PRF CM significantly increased *MMP-13* mRNA levels (Figure 3D), while *TIMP-1* was significantly upregulated by the supplementation of pro-inflammatory cytokines combined with 25% L-PRF CM and DPSC CM compared to the stimulated control after 24 h (Figure 3E). *ADAM-17* mRNA levels were significantly increased upon exposure to cytokines, but not altered by the supplementation of L-PRF ex, L-PRF CM or DPSC CM (Figure 3F). Cytokines with 25% L-PRF CM significantly amplified the *IL-6* mRNA levels compared to the stimulated control (Figure 3G). *TNF-α* and *iNOS* mRNA levels were significantly increased upon exposure to cytokines, and are not altered upon supplementation of L-PRF ex, L-PRF CM or DPSC CM (Figure 3H,I). 

### 3.5. IL-6 and PGE2 Release Are Increased After Supplementation of Cytokines Combined With L-PRF CM

**Figure 4:**
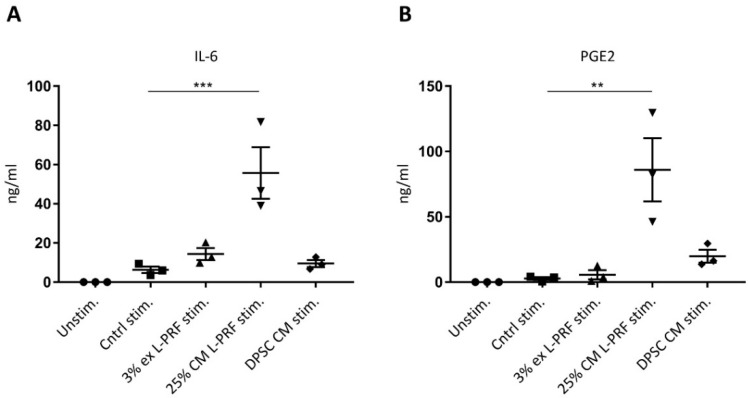
IL-6 and PGE2 secretion of iMACs after exposure to inflammatory cytokines and L-PRF ex, L-PRF CM and DPSC CM, measured via ELISA. (**A**) IL-6 release of iMACs is significantly increased after exposure to cytokine stimulation combined with 25% L-PRF CM. (**B**) Stimulation with cytokines in combination with 25% L-PRF CM induced a significant increase in PGE2 release by iMACs. Data correspond to *n* = 3. Data are represented as mean ± S.E.M. **. *p* ≤ 0.01. ***. *p* ≤ 0.001.

The medium of iMACs cultured in 3% L-PRF ex and 25% L-PRF CM and DPSC CM was collected after 24 h and subjected to an ELISA for IL-6 and PGE2 (Figure 4). Cytokine stimulation enhanced IL-6 production by iMACs, although not significantly, from 0 ng/mL for the unstimulated control to 6.31 ng/mL ± 1.65 ng/mL for the stimulated control (Figure 4A). Of all experimental conditions, only 25% L-PRF CM significantly enhanced IL-6 secretion (Figure 4A). Stimulation with cytokines in combination with 25% L-PRF CM induced a significant increase in PGE2 release by iMACs (86 ng/mL ± 24.14 ng/mL for stimulated 25% L-PRF CM compared to 2.75 ng/mL ± 1.11 ng/mL for the stimulated control) (Figure 4B). 

### 3.6. Nitrite Levels Are Increased Upon Cytokine Stimulation and Decreased by DPSC CM

**Figure 5:**
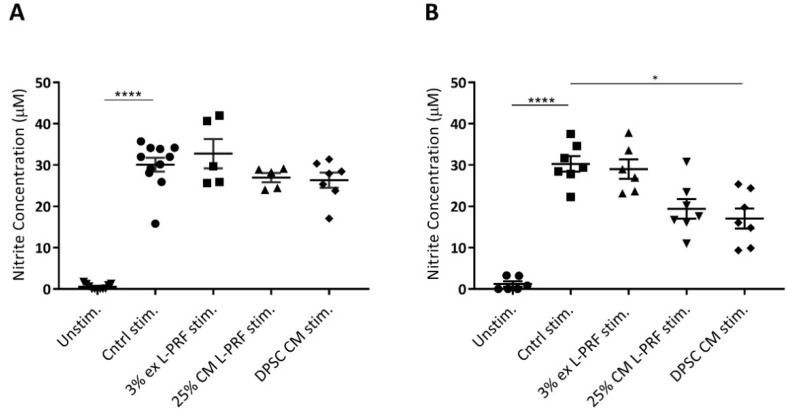
The effect of L-PRF ex, L-PRF CM and DPSC CM on TNF-α and IL-1β-stimulated iMAC nitrite release. Nitrite production in iMACs cultured in monolayer (**A**) and micropellet (**B**) was measured via the Griess assay. (**A**) In monolayer culture, nitrite production was significantly increased upon cytokine stimulation but not significantly altered by exposure to L-PRF ex, L-PRF CM and DPSC CM after 24 h. (**B**) In 3D micropellets, DPSC CM significantly reduced nitrite release of iMACs after 72 h. Data correspond to *n* = 5 for L-PRF ex and L-PRF CM, *n* = 7 for DPSC CM (A), *n* = 6 for L-PRF ex, *n* = 7 for L-PRF CM and DPSC CM (B). Data are represented as mean ± S.E.M. *. *p* ≤ 0.05. ****. *p* ≤ 0.0001.

To evaluate the influence of secreted factors of L-PRF and DPSCs on iMAC nitrite secretion, a Griess assay was performed. iMACs secreted significant more nitrite when they were stimulated with TNF-α and IL-1β in monolayer and 3D pellet culture (Figure 5A,B). In monolayer, 25% L-PRF CM exerted a small decrease in the nitrite secretion from 30.09 μM ± 1.69 μM to 26.69 μM ± 1.13 μM (Figure 5A). However, this effect was not significant. Also in pellet culture, L-PRF CM exerted a small not significant decrease in nitrite release by cytokine-stimulated iMACs. L-PRF ex did not decrease the nitrite secretion in cytokine-stimulated iMAC after 24 h in monolayer, nor after 72 h in pellet culture. CM of DPSCs induced a small, but not significant reduction in nitrite production of iMACs after 24 h from 30.09 μM ± 1.69 μM to 26.33 μM ± 1.84 μM in monolayer (Figure 5A). Remarkably, DPSC CM significantly decreased nitrite secretion of iMACs in micromass culture from 30.25 μM ± 1.87 μM to 17.08 μM ± 2.42 μM (Figure 5B). 

### 3.7. Cartilage-Specific ECM Production of iMACs in 3D Culture After Exposure to L-PRF ex, L-PRF CM and DPSC CM

**Figure 6:**
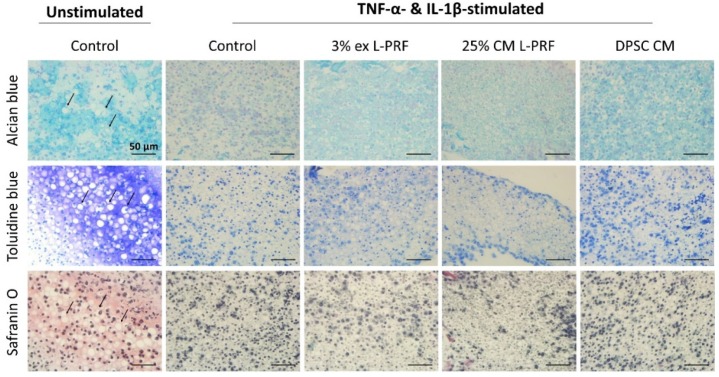
TNF-α- and IL-1β-stimulated iMACs cultured in 3D pellets attenuated a more cartilage-like morphology after exposure to L-PRF ex, L-PRF CM and DPSC CM. Representative images showed that unstimulated iMAC pellets generated a cartilage-like tissue with large numbers of chondrocytes present in lacunae (arrowheads). In the cytokine-stimulated control condition, iMACs developed into a more fibrous tissue in which cartilage-lacunae were less evident and GAG production is meaningfully reduced, as shown by an apparent decrease in alcian blue, toluidine blue and safranin O staining intensity. Cartilage lacunae were more preserved by L-PRF ex, L-PRF CM and DPSC CM with a weak tendency of improved ECM content and chondrocyte status compared to the stimulated control. Data correspond to *n* = 3. Scale bars = 50 μm.

To test the effect of secreted factors of L-PRF and DPSCs on the cartilage-matrix production of cytokine-stimulated iMACs, 5 × 10^5^ iMACs cultured in micromasses were stimulated with TNF-α and IL-1β for 24 h. Afterwards, experimental conditions were added and 72 h later cell pellets were used for histological examination of the cartilaginous structure. Unstimulated iMAC pellets generated a typical cartilage-like tissue composed of chondrocytes in distinct lacunae surrounded by a dense PG-rich matrix as shown by representative images of alcian blue, toluidine blue and safranin O staining (Figure 6, arrowheads). However, pellets formed by cytokine-stimulated iMACs developed into a more fibrous tissue in which cartilage-lacunae were less evident and meaningfully decreased ECM and GAG production could be observed. This was revealed by an obvious decrease in alcian blue, toluidine blue and safranin O staining intensity (Figure 6). Cytokine-stimulated iMACs cultured with 3% L-PRF ex, 25% L-PRF CM and DPSC CM attained slightly more typical cartilage-like lacunae and showed a weak tendency of improved ECM content and chondrocyte status compared to the cytokine-stimulated control. A tendency to a higher alcian blue staining intensity could also be observed when cytokine-stimulated iMACs were cultured on L-PRF ex, L-PRF CM and DPSC CM. 

## 4. Discussion

The suggested mechanisms via which MSCs mediate cartilage repair and aid in OA include replacement of damaged cartilage tissue and paracrine-mediated effects such as proliferation of endogenous cells and immunomodulation [2]. 

In the first phase of the current study, the chondrogenic differentiation capacities of DPSCs were compared to BM-MSCs. Both BM-MSCs and DPSCs were shown to generate compact cartilage-like 3D spheres by differentiated cells surrounded by abundant ECM and GAGs. One of the most predominant PG, aggrecan, was not expressed in differentiated DPSCs, but cartilage spheres generated by BM-MSCs show abundant aggrecan secretion in the ECM. The absence of aggrecan in differentiated DPSC pellets in our study might be ascribed to several factors. One of these factors might be the differentiation time since an improved chondrocyte phenotype is reported upon prolonged culture times [42]. After a differentiation period of 6 weeks, aggrecan expression was reported in human DPSCs by Mata and colleagues [43]. Another factor might be the culture settings as many utilised scaffolds to improve the phenotype of DPSC-derived chondrocytes in vitro, including hydrogels containing poly(ethylene glycol) dimethacrylate (PEGDMA), methacrylated gelatin (GelMA) and hyaluronic acid (HA) [44] and chitosan-based scaffolds [45], but did not always test for aggrecan expression. In addition to differentiation time and culture environment, also hypoxic conditions and the addition of specific carbohydrates or growth factors might improve the expression of cartilage-specific components [24,46,47]. Dai et al. reported that costal chondrocytes combined with exogenous FGF-9 are suitable to supply chondro-inductive stimuli to DPSCs [23]. Rizk and colleagues showed that TGF-β3-transduced DPSCs express chondrogenic markers, including aggrecan [48]. Similar to our data, they showed that a positive staining for aggrecan was not evident in micromasses made by non-transduced DPSCs. Finally, the absence of aggrecan expression in our study might also have been influenced by inter-donor variability, as for example donor age might impact MSC differentiation [49,50]. 

Our data demonstrated that DPSCs show competences to differentiate towards the chondrogenic lineage, however we were not able to show aggrecan expression. Despite other reports indicate that DPSCs could be used for hyaline cartilage engineering studies, these cells might, for example, be promising for engineering of fibrocartilaginous tissues such as for the temporomandibular joint (TMJ). The latter has been demonstrated by a recent report of Longoni et al. in which they show that under various chondro-inductive conditions DPSCs formed more fibrocartilage-like tissues instead of hyaline cartilage [51].

Chondrogenesis of MSCs has been shown to be enhanced by the supplementation of growth factors [24]. The beneficial properties of L-PRF have mainly been attributed to the high concentration of platelets, leukocytes and the long-term release of growth factors by the L-PRF matrix [52]. We investigated the effect of L-PRF ex and L-PRF CM on the chondrogenic differentiation of DPSCs and BM-MSCs. Our results show that L-PRF ex and L-PRF CM were neither able to significantly increase the GAG secretion in both cell types nor induce aggrecan expression in DPSCs. Reports on chondro differentiation-promoting effects of platelets aggregates, such as PRP, on MSCs are contentious. Several previously confirmed chondro-inductive stimuli of platelet concentrates to MSCs [38,53,54,55], whereas others indicate that PRP treatment does not improve the in vitro chondrogenesis of MSCs [56]. The difference between the previously identified differentiation-promoting effects of platelets aggregates such as PRP in musculoskeletal diseases (reviewed by Qian et al. [57]), and our data on L-PRF might be caused by different factors. First, various platelet concentrates have different release kinetics [58]. Second, compared to other platelet concentrates, L-PRF contains significantly higher concentrations of leukocytes [59]. With reference to this, the leukocytes in L-PRF have positive effects (e.g. anti-microbial properties [59]), but might at the same time be involved in catabolic pathways [60]. Moreover, the leukocyte fraction in L-PRF has been reported to be accountable for the overproduction of several growth factors, including VEGF and inflammatory cytokines [59,61], which have been described to negatively impact chondrogenesis in vitro [31,62,63,64,65,66,67]. In contrast, many other growth factors present in L-PRF ex and L-PRF CM are reported to have beneficial influences on MSC chondrogenesis [31,34,68,69]. To date, our data strongly indicate that the supplementation of L-PRF ex and L-PRF CM does not alter MSC chondrogenesis in vitro. 

In a second phase, the secretome-mediated effects of human DPSCs and L-PRF on (TNF-α- and IL-1β-stimulated) iMACs were investigated. iMACs were isolated and phenotypically characterised based upon criteria identified by Gosset et al. [39]. It is broadly documented that chondrocytes de-differentiate to fibroblast-like cells in monolayer and can bias outcomes [39,70]. To overcome this, all data were generated using freshly isolated chondrocytes. TNF-α- and IL-1β-stimulated chondrocytes transformed into cells with a reduced function, such as decreased cartilage-specific matrix mRNA levels, increased MMPs, inflammatory gene expressions and suppressed GAG production. These findings demonstrated the establishment of robust OA-mimicked chondrocytes in vitro.

We demonstrated that L-PRF CM significantly enhanced unstimulated iMAC survival, proliferation and TNF-α- and IL-1β-stimulated iMAC viability in a concentration-dependent manner. These effects were not observed in iMACs cultured in the presence of L-PRF ex. In contrast to our findings, Chien et al. demonstrated that the exudate of PRF could improve chondrocyte proliferation when cultured in fibrin-based scaffolds [35]. This alteration in outcome between the two L-PRF derivatives might be explained in the difference in growth factor levels. Specifically, significantly higher levels of growth factors are found in L-PRF CM compared to L-PRF ex, which can be caused by the fact that L-PRF CM is generated after incubation for 96 h, resulting in a continuous release of growth factors by the leukocytes in the fibrin matrix of the L-PRF [26,31]. RT-qPCR data demonstrated at 24 h post-stimulation a significant decrease in *aggrecan* and *collagen type II α 1* mRNA levels of healthy iMACs when cultured in the presence of L-PRF CM. L-PRF ex significantly decreased *aggrecan* mRNA expression. In addition, *MMP-13* and *TIMP-1* mRNA expressions were increased in unstimulated iMACs upon 25% L-PRF stimulation. The increased proliferative state of iMACs upon L-PRF CM supplementation seems to be accompanied by a downregulation of cartilage-specific ECM components and the upregulation of *MMP-13* in healthy iMACs. When iMACs were cytokine-stimulated, L-PRF CM significantly increased *MMP-13*, *TIMP-1* and *IL-6* mRNA levels. ELISA demonstrated a significant increase of IL-6 and PGE2 secretion, two inflammatory mediators in OA by cytokine-stimulated iMACs upon exposure to 25% L-PRF CM. IL-6 is widely known to mediate several pro-inflammatory responses contributing to the pathogenesis of several immune-related diseases, such as RA [71]. Therefore, therapeutic targeting IL-6 has become important in the drug development applications of these diseases. Tocilizumab (TCZ), an IL-6 receptor-inhibiting monoclonal antibody, is widely used in the treatment of RA [72]. However, the role of IL-6 in OA remains unclear. High levels of IL-6 are found in the synovial fluid of OA patients. These high IL-6 levels are associated with increased MMP levels and radiographic OA changes [73,74]. Additionally, it was reported that inhibition of IL-6 with TCZ lowered pain behaviour in an experimental model of OA in rats [75]. In contrast, IL-6 knockout mice revealed the progression of more advanced OA than wild-type animals and injection of IL-6 in the joint of IL-6-deficient mice reduced cartilage loss during arthritis [76,77]. Nevertheless, based on the above outcomes, our data might indicate an inability of L-PRF to counteract cytokine-induced phenotypical changes of iMACs in vitro.

Several growth factors, such as VEGF, EGF, IL-6 and MCP-1, are highly present in L-PRF CM and in minor levels in the exudate [31,64] and might be accountable for the observed effects in the present study. For example, VEGF is reported to act as a survival factor in growth plate chondrocytes and has proliferative effects in immortalised chondrocytes [78]. Moreover, increased MMP levels and secretion are reported because of VEGF [79,80]. Pufe and colleagues also showed pro-inflammatory factors such as IL-1β, nitric oxide, TNF-α and IL-6 to be induced by VEGF [80]. Also EGF and IL-6 increased numbers of chondrocytes [81,82]. Furthermore, IL-6 is described to be able to increase MMP expression alone or in synergy with IL-1β and oncostatin M [83,84,85]. In addition, MCP-1 increased MMP-13 expression in chondrocytes [86]. Furthermore, several other proteins that are abundantly present in L-PRF CM, such as RANTES, growth regulated oncogene (GRO) and IL-8 might be responsible for the observed effects in our study [31]. RANTES is demonstrated to induce chondrocyte expression of inducible NO synthase, IL-6 and MMP-1 [87], while IL-8 and GROα are shown to induce articular chondrocyte hypertrophy and calcification through increased type X collagen, MMP-13 expression and alkaline phosphatase activity [88]. 

In contrast to our findings, numerous other studies demonstrated that a large number of growth factors found to be secreted by platelet derivatives have predominantly beneficial and promising activities for (pre-)clinical applications for chondrogenesis and anti-inflammatory effects [33]. To date, studies mainly focused on the role of platelets and platelet-derived growth factors, since these are the common features between all types of platelet concentrates, while future research should focus on identifying the role of the leukocytes and leukocyte-derived growth factors and cytokines in L-PRF. To our knowledge, reports on the secretome-mediated effects of L-PRF on chondrocytes in vitro are limited. Injectable-PRF, generated by a low speed centrifugation approach, was found to counteract IL-1β inflammatory effects in chondrocytes [36]. In addition, Wong et al. treated chondrocytes with different concentrations of PRF CM and showed a proliferative effect on chondrocytes and induced chondrogenic differentiation of chondrocytes [37]. Moreover, Barbon et al. revealed that pre-clinical studies strongly indicate a significant enhancement of cartilage regeneration after PRF treatment [34]. There are several reasons for the discrepancy in outcomes between our study and studies proving beneficial effects of PRP or PRF in OA. One crucial factor might the inter-donor variability. Second, as previously mentioned, platelet concentrates differ in growth factor kinetics, release levels and amount of leukocytes. In addition, absolute platelet, white blood cell and red blood cell concentrations can vary in different preparations. 

In the present study, we show that the CM of DPSCs significantly enhances iMAC survival and proliferation in vitro. DPSC CM exerts the same, but smaller, effects on TNF-α and IL-1β-stimulated iMAC viability although not reaching statistical significance. DPSCs secrete various growth factors and cytokines, which might be accountable for the observed outcomes in the present study. Previous studies revealed high expression levels of TGFs and neurotrophic factors, including VEGF [20,89,90]. Other factors present in the DPSC secretome involve but are not limited to IL-8, MCP-1, FGFs, MMPs, TIMP-1 [18,20,91]. The presence of large quantities of VEGF in DPSCs could predominantly be responsible for the proliferative effects on iMACs [78]. Narcisi et al. report that TGF-β1-stimulated chondrocytes evidenced increased mRNA levels for several hypertrophy-specific markers, including *MMP-13*, *VEGF* and *TIMP-3* [92]. In our study, RT-qPCR data show that DPSC CM induced significantly increased *TIMP-1* expression in stimulated iMACs. TIMP-1 directly inhibits the activities of MMPs, thereby contributing to reducing the impact of MMPs [93,94]. Next to this, in cytokine-stimulated chondrocytes cultured on DPSC CM a not significant trend towards increased PGE2 production was observed. The role of PGE2 is controversial in OA; though PGE2 exerts catabolic functions in OA, one of the main effectors of MSC-mediated immune-suppression is PGE2 [95]. 

Cartilage-specific ECM production of iMACs in 3D culture after exposure to the secretome of L-PRF and DPSCs was also evaluated. The benefit of using these micromass cultures compared to monolayer cultures is that the 3D setting is more representative of the in vivo microenvironment. In consistence with our RT-qPCR results, cytokine stimulation of iMACs induced meaningfully less production of PGs and GAGs in micromass cultures, accompanied by increased nitrite secretion. Cartilage lacunae were more preserved by 3% L-PRF ex, 25% L-PRF CM and DPSC CM with a weak tendency of improved ECM content and chondrocyte status as compared to the stimulated control. Moreover, DPSC CM significantly decreased nitrite levels of iMACs cultured in 3D micromasses. It should be noted that the experiments in this study were conducted between 24–72 h post-stimulation, a time window in which the outcome on matrix components like aggrecan or collagen type II production is not yet observed. Therefore, we also evaluated fast acting proteins such as nitrite and PGE2. Nonetheless, subsequent studies are necessary to investigate the impact on the structural level of cartilage by means of longer in vitro cultures, cartilage-explant studies and in vivo experiments. Given the pathophysiology of OA, the role of immune cells, other cell types present in the synovial joint and synovial joint structures should ideally also be taken into account. Therefore, in order to supply a proper in vitro OA model, the interchange of immune cells, the synovial membrane and subchondral bone with the cartilage tissue and chondrocytes needs to be addressed. One-dimensional cell culture models cannot fully mimic the complexity of the OA pathophysiology. However, several advantages are associated with monolayer or one-dimensional cell cultures such as a large number of cells can be easily isolated, and cells in monolayer permit the homogenous spread of cytokines and nutrients. Still, co-cultures or 3D cultures permit the study of cell-specific changes and cell–cell communications, while explant models inform on the induced alterations occurring in the ECM. The co-culture of the synovium with chondrocytes is one way to reproduce the complexity of the pro-inflammatory events in vitro. The use of bone in co-culture experiments is also crucial [96]. Haltmayer et al. utilised a co-culture system with all three principal tissues involved in OA, such as cartilage, subchondral bone and the synovium [97].

## 5. Conclusions

The present study aimed to investigate the chondrogenic potential of both L-PRF and DPSCs in vitro in terms of being able to replace lost cartilage tissue, while having chondroprotective and immunomodulatory influences in OA chondrocytes. We show a discrepancy between BM-MSCs and DPSCs to form neo hyaline cartilage in vitro and that L-PRF did not improve or impede the chondrogenic differentiation of both DPSCs and BM-MSCs. However, DPSCs generated a GAG- and collagen-rich matrix, demonstrating that DPSCs are a promising cell source to make cartilage regeneration achievable. L-PRF CM exerted significant pro-survival and proliferative effects on chondrocytes and increased several inflammation-related mediators involved in OA. Nevertheless, transformation into hypertrophic chondrocytes remains an important matter that needs to be further elucidated. Our data show promising therapeutic effects of DPSCs to repair cartilage lesions and in an in vitro model mimicking OA, as they can potentially replace the damaged cartilage tissue and act via secretome-mediated effects. On the one hand, DPSC CM can stimulate endogenous cells to proliferate and replace the lost tissue, while on the other hand, it could prevent the progression of cartilage loss by impairing chondrocyte apoptosis. Moreover, we indicate that factors secreted by DPSCs might cause multiple anti-inflammatory and anti-catabolic influences in OA chondrocytes. Insights in the paracrine effects of DPSCs and understanding stem cell modulation will offer researchers a number of treatment options for musculoskeletal diseases and traumatic injury that have until now been limited by cell sourcing concerns. Finally, the influence of secretome-mediated actions of L-PRF and DPSCs on OA chondrocytes and other types of cells or joint structures involved in OA should additionally be investigated in longer-term co-culture systems or 3D cell culture settings. Furthermore, since hypertrophic chondrocytes are important in pathological modifications in OA, a future study to investigate the dedifferentiated or hypertrophic state of chondrocytes is warranted.

## Supplementary Materials

The following are available online at https://www.mdpi.com/2073-4409/9/4/980/s1, Supplementary Materials and Methods, Table S1: The Primers used for RT-qPCR analysis. Figure S1: The effect of L-PRF ex, L-PRF CM and DPSC CM on chondrogenic genes of iMACs.

## References

[1] Fox A.J.S., Bedi A., Rodeo S.A. (2009). The Basic Science of Articular Cartilage. Sports Heal. Multidiscip. Approach.

[2] Monaco M.L., Merckx G., Ratajczak J., Gervois P., Hilkens P., Clegg P.D., Bronckaers A., Vandeweerd J.-M., Lambrichts I. (2018). Stem Cells for Cartilage Repair: Preclinical Studies and Insights in Translational Animal Models and Outcome Measures. Stem Cells Int..

[3] Solheim E., Krokeide A.M., Melteig P., Larsen A., Strand T., Brittberg M. (2014). Symptoms and function in patients with articular cartilage lesions in 1000 knee arthroscopies. Knee Surg. Sports Traumatol. Arthrosc..

[4] Widuchowski W., Widuchowski J., Koczy B., Szyluk K. (2009). Untreated Asymptomatic Deep Cartilage Lesions Associated with Anterior Cruciate Ligament Injury. Am. J. Sports Med..

[5] Widuchowski W., Widuchowski J., Faltus R., Lukasik P., Kwiatkowski G., Szyluk K., Koczy B. (2011). Long-term clinical and radiological assessment of untreated severe cartilage damage in the knee: A natural history study. Scand. J. Med. Sci. Sports.

[6] Brooks P.M. (2002). Impact of osteoarthritis on individuals and society: How much disability? Social consequences and health economic implications. Curr. Opin. Rheumatol..

[7] Litwic A., Edwards M., Dennison E.M., Cooper C. (2013). Epidemiology and burden of osteoarthritis. Br. Med. Bull..

[8] World Health Organization https://www.who.int/chp/topics/rheumatic/en/.

[9] Alshami A.M. (2014). Knee osteoarthritis related pain: A narrative review of diagnosis and treatment. Int. J. Heal. Sci..

[10] Negoro T., Takagaki Y., Okura H., Matsuyama A. (2018). Trends in clinical trials for articular cartilage repair by cell therapy. NPJ Regen. Med..

[11] Pareek A., Carey J.L., Reardon P.J., Peterson L., Stuart M.J., Krych A.J. (2016). Long-Term Outcomes after Autologous Chondrocyte Implantation. Cartilage.

[12] Niemeyer P., Albrecht D., Andereya S., Angele P., Ateschrang A., Aurich M., Baumann M., Bosch U., Erggelet C., Fickert S. (2016). Autologous chondrocyte implantation (ACI) for cartilage defects of the knee: A guideline by the working group “Clinical Tissue Regeneration” of the German Society of Orthopaedics and Trauma (DGOU). Knee.

[13] Aae T.F., Randsborg P.-H., Lurås H., Årøen A., Lian Øystein B. (2017). Microfracture is more cost-effective than autologous chondrocyte implantation: A review of level 1 and level 2 studies with 5 year follow-up. Knee Surg. Sports Traumatol. Arthrosc..

[14] Shi S., Gronthos S. (2003). Perivascular Niche of Postnatal Mesenchymal Stem Cells in Human Bone Marrow and Dental Pulp. J. Bone Miner. Res..

[15] Ibarretxe G., Crende O., Aurrekoetxea M., García-Murga V., Etxaniz J., Unda F. (2012). Neural Crest Stem Cells from Dental Tissues: A New Hope for Dental and Neural Regeneration. Stem Cells Int..

[16] Ledesma-Martínez E., Mendoza-Núñez V.M., Santiago-Osorio E. (2015). Mesenchymal Stem Cells Derived from Dental Pulp: A Review. Stem Cells Int..

[17] Hilkens P., Gervois P., Fanton Y., Vanormelingen J., Martens W., Struys T., Politis C., Lambrichts I., Bronckaers A. (2013). Effect of isolation methodology on stem cell properties and multilineage differentiation potential of human dental pulp stem cells. Cell and Tissue Research.

[18] Ahmed N., Murakami M., Hirose Y., Nakashima M. (2016). Therapeutic Potential of Dental Pulp Stem Cell Secretome for Alzheimer’s Disease Treatment: An In Vitro Study. Stem Cells Int..

[19] Hossein-Khannazer N., Hashemi S.M., Namaki S., Ghanbarian H., Sattari M., Khojasteh A. (2019). Study of the immunomodulatory effects of osteogenic differentiated human dental pulp stem cells. Life Sci..

[20] Bronckaers A., Hilkens P., Fanton Y., Struys T., Gervois P., Politis C., Martens W., Lambrichts I. (2013). Angiogenic Properties of Human Dental Pulp Stem Cells. PLoS ONE.

[21] Li Z., Jiang C.-M., An S., Cheng Q., Huang Y.-F., Wang Y.-T., Gou Y., Xiao L., Yu W.-J., Wang J. (2013). Immunomodulatory properties of dental tissue-derived mesenchymal stem cells. Oral Dis..

[22] Ishikawa J., Takahashi N., Matsumoto T., Yoshioka Y., Yamamoto N., Nishikawa M., Hibi H., Ishigro N., Ueda M., Furukawa K. (2016). Factors secreted from dental pulp stem cells show multifaceted benefits for treating experimental rheumatoid arthritis. Bone.

[23] Dai J., Wang J., Lu J., Zou D., Sun H., Dong Y., Yu H., Zhang L., Yang T., Zhang X. (2012). The effect of co-culturing costal chondrocytes and dental pulp stem cells combined with exogenous FGF9 protein on chondrogenesis and ossification in engineered cartilage. Biomaterials.

[24] Fortier L.A., Barker J.U., Strauss E.J., McCarrel T., Cole B.J. (2011). The Role of Growth Factors in Cartilage Repair. Clin. Orthop. Relat. Res..

[25] Sampson S., Gerhardt M., Mandelbaum B. (2008). Platelet rich plasma injection grafts for musculoskeletal injuries: A review. Curr. Rev. Musculoskelet. Med..

[26] Ehrenfest D.M.D., Rasmusson L., Albrektsson T. (2009). Classification of platelet concentrates: From pure platelet-rich plasma (P-PRP) to leucocyte- and platelet-rich fibrin (L-PRF). Trends Biotechnol..

[27] Kennedy M.I., Whitney K., Evans T., Laprade R.F. (2018). Platelet-Rich Plasma and Cartilage Repair. Curr. Rev. Musculoskelet. Med..

[28] Moussa M., Lajeunesse D., Hilal G., El Atat O., Haykal G., Serhal R., Chalhoub A., Khalil C., Alaaeddine N. (2017). Platelet rich plasma (PRP) induces chondroprotection via increasing autophagy, anti-inflammatory markers, and decreasing apoptosis in human osteoarthritic cartilage. Exp. Cell Res..

[29] Akeda K., An H., Okuma M., Attawia M., Miyamoto K., Thonar E.-M., Lenz M., Sah R., Masuda K. (2006). Platelet-rich plasma stimulates porcine articular chondrocyte proliferation and matrix biosynthesis. Osteoarthr. Cartil..

[30] Prakash S., Thakur A. (2011). Platelet Concentrates: Past, Present and Future. J. Maxillofac. Oral Surg..

[31] Ratajczak J., Vangansewinkel T., Gervois P., Merckx G., Hilkens P., Quirynen M., Lambrichts I., Bronckaers A. (2018). Angiogenic Properties of ‘Leukocyte- and Platelet-Rich Fibrin’. Sci. Rep..

[32] De Melo B.A.G., Luzo Ângela C.M., Lana J.F.S.D. (2019). Centrifugation Conditions in the L-PRP Preparation Affect Soluble Factors Release and Mesenchymal Stem Cell Proliferation in Fibrin Nanofibers. Molecules.

[33] Kabiri A., Esfandiari E., Esmaeili A., Hashemibeni B., Pourazar A., Mardani M. (2014). Platelet-rich plasma application in chondrogenesis. Adv. Biomed. Res..

[34] Barbon S., Stocco E., Macchi V., Contran M., Grandi C., Borean A., Parnigotto P.P., Porzionato A., De Caro R. (2019). Platelet-Rich Fibrin Scaffolds for Cartilage and Tendon Regenerative Medicine: From Bench to Bedside. Int. J. Mol. Sci..

[35] Chien C.-S., Ho H.-O., Liang Y.-C., Ko P.-H., Sheu M.-T., Chen C.-H. (2012). Incorporation of exudates of human platelet-rich fibrin gel in biodegradable fibrin scaffolds for tissue engineering of cartilage. J. Biomed. Mater. Res. Part B Appl. Biomater..

[36] El Raouf M.A., Wang X., Miusi S., Chai J., Abdel-Aal A.B.M., Helmy M.M.N., Ghanaati S., Choukroun J., Choukroun E., Zhang Y. (2017). Injectable-platelet rich fibrin using the low speed centrifugation concept improves cartilage regeneration when compared to platelet-rich plasma. Platelets.

[37] Wong C.-C., Chen C.-H., Chan W.P., Chiu L.-H., Ho W.-P., Hsieh F.-J., Chen Y.-T., Yang T.-L. (2017). Single-Stage Cartilage Repair Using Platelet-Rich Fibrin Scaffolds With Autologous Cartilaginous Grafts. Am. J. Sports Med..

[38] De Souza F.G., Fernandes B., Rebelatto C.L.K., De Aguiar A.M., Fracaro L., Brofman P.R.S. (2018). Proliferation and differentiation of stem cells in contact with eluate from fibrin-rich plasma membrane. Rev. Bras. de Ortop. (Engl. Ed.).

[39] Gosset M., Berenbaum F., Thirion S., Jacques C. (2008). Primary culture and phenotyping of murine chondrocytes. Nat. Protoc..

[40] Gervois P., Struys T., Hilkens P., Bronckaers A., Ratajczak J., Politis C., Brone B., Lambrichts I., Martens W. (2015). Neurogenic Maturation of Human Dental Pulp Stem Cells Following Neurosphere Generation Induces Morphological and Electrophysiological Characteristics of Functional Neurons. Stem Cells Dev..

[41] Bretschneider A., Burns W., Morrison A. (1981). Pop-Off Technique—he Ultrastructure of Paraffin-Embedded Sections. Am J Clin Pathol.

[42] Branly T., Contentin R., Desancé M., Jacquel T., Bertoni L., Leroy S., Mallein-Gerin F., Denoix J.-M., Audigié F., Demoor M. (2018). Improvement of the Chondrocyte-Specific Phenotype upon Equine Bone Marrow Mesenchymal Stem Cell Differentiation: Influence of Culture Time, Transforming Growth Factors and Type I Collagen siRNAs on the Differentiation Index. Int. J. Mol. Sci..

[43] Mata M., Milian L., Oliver M., Zurriaga J., Sancho-Tello M., De Llano J.J.M., Carda C. (2017). In Vivo Articular Cartilage Regeneration Using Human Dental Pulp Stem Cells Cultured in an Alginate Scaffold: A Preliminary Study. Stem Cells Int..

[44] Nemeth C.L., Janebodin K., Yuan A.E., Dennis J.E., Reyes M., Kim D.-H. (2014). Enhanced Chondrogenic Differentiation of Dental Pulp Stem Cells Using Nanopatterned PEG-GelMA-HA Hydrogels. Tissue Eng. Part A.

[45] Westin C., Trinca R.B., Zuliani C., Coimbra I.B., Moraes Â.M. (2017). Differentiation of dental pulp stem cells into chondrocytes upon culture on porous chitosan-xanthan scaffolds in the presence of kartogenin. Mater. Sci. Eng. C.

[46] Christiansen-Weber T., Noskov A., Cardiff D., Garitaonandia I., Dillberger A., Semechkin A., Gonzalez R., Kern R. (2018). Supplementation of specific carbohydrates results in enhanced deposition of chondrogenic-specific matrix during mesenchymal stem cell differentiation. J. Tissue Eng. Regen. Med..

[47] Choi J.R., Pingguan-Murphy B., Abas W.A.B.W., Azmi M.A.N., Omar S.Z., Chua K.H., Safwani W.K.Z.W. (2014). Impact of low oxygen tension on stemness, proliferation and differentiation potential of human adipose-derived stem cells. Biochem. Biophys. Res. Commun..

[48] Rizk A., Rabie A.B.M. (2013). Human dental pulp stem cells expressing transforming growth factor β3 transgene for cartilage-like tissue engineering. Cytotherapy.

[49] Khan H., Mafi P., Mafi R., Khan W.S. (2016). The effects of ageing on differentiation and characterisation of human mesenchymal stem cells. Curr. Stem Cell Res. Ther..

[50] Mohamed-Ahmed S., Fristad I., Lie S., Suliman S., Mustafa K., Vindenes H., Idris S.B. (2018). Adipose-derived and bone marrow mesenchymal stem cells: A donor-matched comparison. Stem Cell Res. Ther..

[51] Longoni A., Utomo L., Van Hooijdonk I., Bittermann G., Vetter V.C., Spanjer E.C.K., Ross J., Rosenberg A., Gawlitta D. (2020). The chondrogenic differentiation potential of dental pulp stem cells. Eur. Cells Mater..

[52] Pinto N.R., Ubilla M., Zamora Y., Del Rio V., Ehrenfest D.M.D., Quirynen M. (2017). Leucocyte- and platelet-rich fibrin (L-PRF) as a regenerative medicine strategy for the treatment of refractory leg ulcers: A prospective cohort study. Platelets.

[53] Mardani M., Kabiri A., Esfandiari E., Esmaeili A., Pourazar A., Ansar M., Hashemibeni B. (2013). The Effect of Platelet Rich Plasma on Chondrogenic Differentiation of Human Adipose Derived Stem Cells in Transwell Culture. Iran. J. Basic Med. Sci..

[54] Drengk A., Zapf A., Stürmer E.K., Stürmer K.M., Frosch K.-H. (2009). Influence of Platelet-Rich Plasma on Chondrogenic Differentiation and Proliferation of Chondrocytes and Mesenchymal Stem Cells. Cells Tissues Organs.

[55] Mishra A., Tummala P., King A., Lee B., Kraus M., Tse V., Jacobs C.R. (2009). Buffered Platelet-Rich Plasma Enhances Mesenchymal Stem Cell Proliferation and Chondrogenic Differentiation. Tissue Eng. Part C Methods.

[56] Liou J.-J., Rothrauff B., Alexander P.G., Tuan R.S. (2018). Effect of Platelet-Rich Plasma on Chondrogenic Differentiation of Adipose- and Bone Marrow-Derived Mesenchymal Stem Cells. Tissue Eng. Part A.

[57] Qian Y., Han Q., Chen W., Song J., Zhao X., Ouyang Y., Yuan W.-E., Fan C. (2017). Platelet-Rich Plasma Derived Growth Factors Contribute to Stem Cell Differentiation in Musculoskeletal Regeneration. Front. Chem..

[58] Kobayashi E., Flückiger L., Fujioka-Kobayashi M., Sawada K., Sculean A., Schaller B., Miron R. (2016). Comparative release of growth factors from PRP, PRF, and advanced-PRF. Clin. Oral Investig..

[59] Bielecki T., Ehrenfest D.M.D., Everts P.A., Wiczkowski A. (2012). The role of leukocytes from L-PRP/L-PRF in wound healing and immune defense: New perspectives. Curr. Pharm. Biotechnol..

[60] Ozer K., Colak O. (2019). Leucocyte- and platelet-rich fibrin as a rescue therapy for small-to-medium-sized complex wounds of the lower extremities. Burn. Trauma.

[61] Ehrenfest D.M.D., De Peppo G.M., Doglioli P., Sammartino G. (2009). Slow release of growth factors and thrombospondin-1 in Choukroun’s platelet-rich fibrin (PRF): A gold standard to achieve for all surgical platelet concentrates technologies. Growth Factors.

[62] Kubo S., Cooper G.M., Matsumoto T., Phillippi J.A., Corsi K.A., Usas A., Li G., Fu F.H., Huard J. (2009). Blocking vascular endothelial growth factor with soluble Flt-1 improves the chondrogenic potential of mouse skeletal muscle-derived stem cells. Arthritis Rheum..

[63] Yoon Y.-M. (2000). Epidermal Growth Factor Negatively Regulates Chondrogenesis of Mesenchymal Cells by Modulating the Protein Kinase C-alpha, Erk-1, and p38 MAPK Signaling Pathways. J. Boil. Chem..

[64] Gervois P., Ratajczak J., Wolfs E., Vangansewinkel T., Dillen Y., Merckx G., Bronckaers A., Lambrichts I. (2019). Preconditioning of Human Dental Pulp Stem Cells with Leukocyte- and Platelet-Rich Fibrin-Derived Factors Does Not Enhance Their Neuroregenerative Effect. Stem Cells Int..

[65] Wehling N., Palmer G.D., Pilapil C., Liu F., Wells J.W., Müller P.E., Evans C.H., Porter R.M. (2009). Interleukin-1β and tumor necrosis factor α inhibit chondrogenesis by human mesenchymal stem cells through NF-κB-dependent pathways. Arthritis Rheum..

[66] Liu W., Sun Y., He Y., Zhang H., Zheng Y., Yao Y., Zhang Z. (2016). IL-1β impedes the chondrogenic differentiation of synovial fluid mesenchymal stem cells in the human temporomandibular joint. Int. J. Mol. Med..

[67] Zayed M., Schumacher J., Misk N.A., Dhar M. (2016). Effects of pro-inflammatory cytokines on chondrogenesis of equine mesenchymal stromal cells derived from bone marrow or synovial fluid. Veter- J..

[68] Danisovic L., Varga I., Polak S. (2012). Growth factors and chondrogenic differentiation of mesenchymal stem cells. Tissue Cell.

[69] Yang A., Lu Y., Xing J., Li Z., Yin X., Dou C., Dong S., Luo F., Xie Z., Hou T. (2018). IL-8 Enhances Therapeutic Effects of BMSCs on Bone Regeneration via CXCR2-Mediated PI3k/Akt Signaling Pathway. Cell. Physiol. Biochem..

[70] Stokes D.G., Liu G., Coimbra I.B., Piera-Velazquez S., Crowl R.M., A Jimenez S. (2002). Assessment of the gene expression profile of differentiated and dedifferentiated human fetal chondrocytes by microarray analysis. Arthritis Rheum..

[71] Srirangan S., Choy E.H. (2010). The Role of Interleukin 6 in the Pathophysiology of Rheumatoid Arthritis. Ther. Adv. Musculoskelet. Dis..

[72] Ogata A., Kato Y., Higa S., Yoshizaki K. (2019). IL-6 inhibitor for the treatment of rheumatoid arthritis: A comprehensive review. Mod. Rheumatol..

[73] Stannus O., Jones G., Cicuttini F., Parameswaran V., Quinn S., Burgess J., Ding C. (2010). Circulating levels of IL-6 and TNF-α are associated with knee radiographic osteoarthritis and knee cartilage loss in older adults. Osteoarthr. Cartil..

[74] Vuolteenaho K., Koskinen-Kolasa A., Laavola M., Nieminen R., Moilanen T., Moilanen E. (2017). High synovial fluid interleukin-6 levels are associated with increased matrix metalloproteinase levels and severe radiographic changes in osteoarthritis patients. Osteoarthr. Cartil..

[75] Lin Y., Liu L., Jiang H., Zhou J., Tang Y. (2017). Inhibition of interleukin-6 function attenuates the central sensitization and pain behavior induced by osteoarthritis. Eur. J. Pharmacol..

[76] Van De Loo F.A., Kuiper S., Van Enckevort F.H., Arntz O.J., Berg W.B.V.D. (1997). Interleukin-6 reduces cartilage destruction during experimental arthritis. A study in interleukin-6-deficient mice. Am. J. Pathol..

[77] De Hooge A.S., Van De Loo F.A., Bennink M.B., Arntz O.J., De Hooge P., Berg W.B.V.D., Vandenberg W. (2005). Male IL-6 gene knock out mice developed more advanced osteoarthritis upon aging. Osteoarthr. Cartil..

[78] Nagao M., Hamilton J.L., Kc R., Berendsen A.D., Duan X., Cheong C., Li X., Im H.-J., Olsen B.R. (2017). Vascular Endothelial Growth Factor in Cartilage Development and Osteoarthritis. Sci. Rep..

[79] Enomoto H., Inoki I., Komiya K., Shiomi T., Ikeda E., Obata K.-I., Matsumoto H., Toyama Y., Okada Y. (2003). Vascular Endothelial Growth Factor Isoforms and Their Receptors Are Expressed in Human Osteoarthritic Cartilage. Am. J. Pathol..

[80] Pufe T., Harde V., Petersen W., Goldring M.B., Tillmann B., Mentlein R. (2004). Vascular endothelial growth factor(VEGF) induces matrix metalloproteinase expression in immortalized chondrocytes. J. Pathol..

[81] Klooster A.R., Bernier S.M. (2004). Tumor necrosis factor alpha and epidermal growth factor act additively to inhibit matrix gene expression by chondrocyte. Arthritis Res. Ther..

[82] Namba A., Aida Y., Suzuki N., Watanabe Y., Kawato T., Motohashi M., Maeno M., Matsumura H., Matsumoto M. (2007). Effects of IL-6 and Soluble IL-6 Receptor on the Expression of Cartilage Matrix Proteins in Human Chondrocytes. Connect. Tissue Res..

[83] Zanotti S., Canalis E. (2013). Interleukin 6 mediates selected effects of Notch in chondrocytes. Osteoarthr. Cartil..

[84] Cawston T.E., Curry V.A., Summers C.A., Clark I.M., Riley G.P., Life P.F., Spaull J.R., Goldring M.B., Koshy P.J., Rowan A.D. (1998). The role of oncostatin M in animal and human connective tissue collagen turnover and its localization within the rheumatoid joint. Arthritis Rheum.

[85] Rowan A.D., Koshy P.J.T., Shingleton W., Degnan B.A., Heath J.K., Vernallis A.B., Spaull J.R., Life P.F., Hudson K., Cawston T.E. (2001). Synergistic effects of glycoprotein 130 binding cytokines in combination with interleukin-1 on cartilage collagen breakdown. Arthritis Rheum..

[86] Xu Y.-K., Ke Y., Wang B., Lin J.-H. (2015). The role of MCP-1-CCR2 ligand-receptor axis in chondrocyte degradation and disease progress in knee osteoarthritis. Boil. Res..

[87] Alaaeddine N., Olee T., Hashimoto S., Creighton-Achermann L., Lotz M. (2001). Production of the chemokine RANTES by articular chondrocytes and role in cartilage degradation. Arthritis Rheum..

[88] Merz D., Liu R., Johnson K.A., Terkeltaub R. (2003). IL-8/CXCL8 and Growth-Related Oncogene α/CXCL1 Induce Chondrocyte Hypertrophic Differentiation. J. Immunol..

[89] Kumar A., Kumar V., Rattan V., Jha V., Bhattacharyya S. (2016). Secretome Cues Modulate the Neurogenic Potential of Bone Marrow and Dental Stem Cells. Mol. Neurobiol..

[90] Man R.C., Sulaiman N., Idrus R., Ariffin S.H.Z., Wahab R.M.A., Yazid M.D. (2019). Insights into the Effects of the Dental Stem Cell Secretome on Nerve Regeneration: Towards Cell-Free Treatment. Stem Cells Int..

[91] Tran-Hung L., Laurent P., Camps J., About I. (2008). Quantification of angiogenic growth factors released by human dental cells after injury. Arch. Oral Boil..

[92] Narcisi R., Quarto R., Ulivi V., Muraglia A., Molfetta L., Giannoni P. (2012). TGFβ-1 administration during Ex vivo expansion of human articular chondrocytes in a serum-free medium redirects the cell phenotype toward hypertrophy. J. Cell. Physiol..

[93] Lozito T.P., Tuan R.S. (2010). Mesenchymal stem cells inhibit both endogenous and exogenous MMPs via secreted TIMPs. J. Cell. Physiol..

[94] Brew K., Nagase H. (2010). The tissue inhibitors of metalloproteinases (TIMPs): An ancient family with structural and functional diversity. Biochim. Biophys. Acta (BBA) Bioenerg..

[95] Mancuso P., Raman S., Glynn A., Barry F., Murphy M. (2019). Mesenchymal Stem Cell Therapy for Osteoarthritis: The Critical Role of the Cell Secretome. Front. Bioeng. Biotechnol..

[96] Johnson C.I., Argyle D.J., Clements D.N. (2016). In vitro models for the study of osteoarthritis. Veter- J..

[97] Haltmayer E., Ribitsch I., Gabner S., Rosser J., Gueltekin S., Peham J., Giese U., Dolezal M., Egerbacher M., Jenner F. (2019). Co-culture of osteochondral explants and synovial membrane as in vitro model for osteoarthritis. PLoS ONE.

