# Supplementary material for: Therapeutic Potential of Dental Pulp Stem Cells and Leukocyte- and Platelet-Rich Fibrin for Osteoarthritis
Source: Cells. 2020 Apr 15;9(4):980. doi: 10.3390/cells9040980 (PMC7227024; doi:10.3390/cells9040980)
Supplement: Supplementary file 1 [file cells-09-00980-s001.pdf]

Supplemental Materials and Methods

Safranin O

Samples were incubated with haematoxylin for 8 min, and washed with running tap water for 20 min. Next, they were incubated with 0.05% Fast Green solution for 5 min and rinsed with 1% acetic acid solution for 10 seconds. Thereafter, the samples were incubated in 0.1% safranin O solution (Merck, Overijse, Belgium) for 5 min.

Alcian blue

Samples were incubated with alcian blue solution (pH = 2.5) for 30 min at room temperature (RT). Subsequently, samples were washed with running tap water for 10 min and submerged in distilled water for 1 min. Next, nuclear fast red solution was applied for 10 min and samples were dipped for 1 second in distilled water.

Toluidine blue

Samples were incubated with 1% toluidine blue solution for 20 min at RT. Subsequently, samples were washed with distilled water for 30 seconds.

Masson's trichrome

After incubation with haematoxylin and running tap water, samples were incubated in Ponceau/Fuchsine solution for 5 min. Next, samples were incubated in 1% phosphomolybdic acid and Aniline blue solution for 5 min each. After incubation in 1% phosphomolybdic acid for 5 min, samples were placed in acetic acid for 2 min. Between each incubation, samples were washed with distilled water.

Primer sequences used for RT-qPCR

Table S1. The Primers used for RT-qPCR analysis.

Gene	Forward primer 5′-3′	Reverse primer 5′-3′
ADAM-17	AGAGAGCCATCTGAAGAGTTTGT	CTTCTCCACGGCCCATGTAT
ACAN	GTCGCTCCCCAACTATCCAG	AAAGTCCAGGGTGTAGCGTG
COL IIα1	GAAGGATGGCTGCACGAAAC	AATAATGGGAAGGCGGGAGG
CYPA	GCGTCTCCTTCGAGCTGTT	AAGTCACCACCCTGGCA
HMBS	GATGGGCAACTGTACCTGACTG	CTGGGCTCCTCTTGGAATG
HPRT	CTCATGGACTGATTATGGACAGGAC	GCAGGTCAGCAAAGAACTTATAGCC
IL-6	TACCACTTCACAAGTCGGAGGC	CTGCAAGTGCATCATCGTTGTTC
iNOS	CCCTTCAATGGTTGGTACATGG	ACATTGATCTCCGTGACAGCC
MMP-13	TCGCCCTTTTGAGACCACTC	AGCACCAAGTGTTACTCGCT
TGF-β	GGGCTACCATGCCAACTTCTG	GAGGGCAAGGACCTTGCTGTA
TIMP-1	TCCTAGAGACACACCAGAGCA	AGCAACAAGAGGATGCCAGA
TNF-α	GTCCCCAAAGGGATGAGAAGT	TTTGCTACGACGTGGGCTAC
YWHAZ	GCAACGATGTACTGTCTCTTTTGG	GTCCACAATTCCTTTCTTGTCATC

Supplemental Figures

Figure S1.

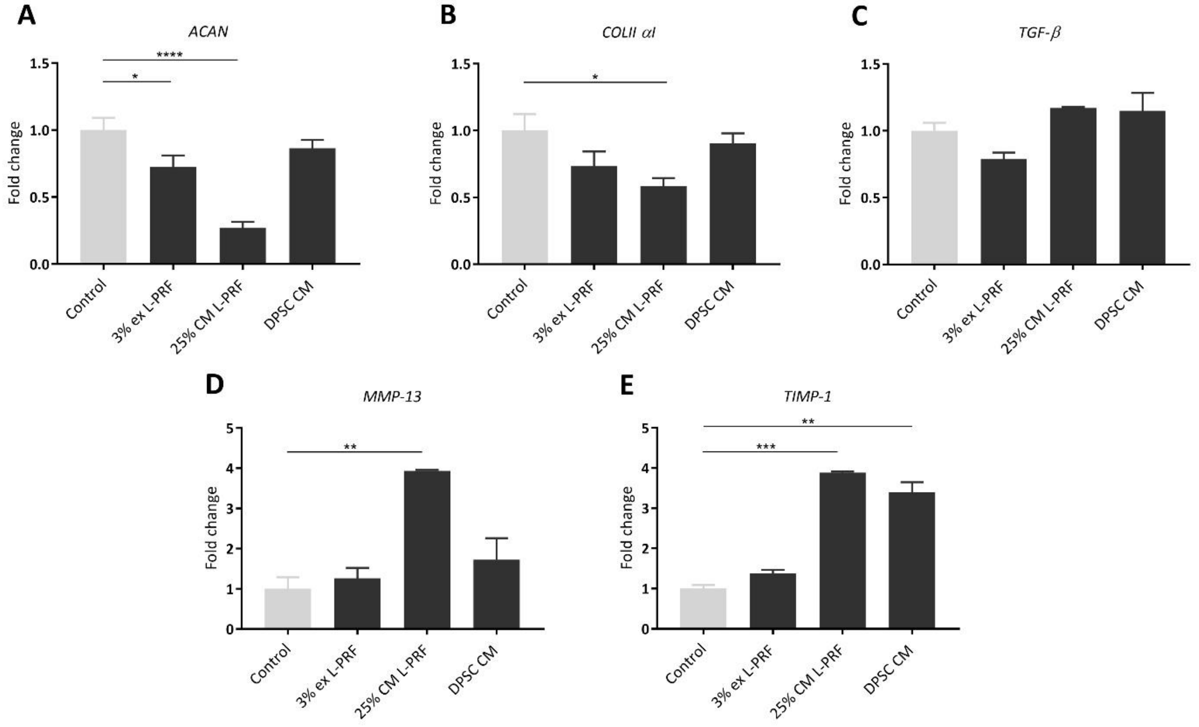

Figure S1. The effect of L-PRF ex, L-PRF CM and DPSC CM on chondrogenic genes of iMACs. Gene expression levels of chondrogenic markers were determined by RT-qPCR of unstimulated iMACs exposed to 3% L-PRF ex, 25% L-PRF CM and DPSC CM. (**A-B**) 25% L-PRF CM significantly decreased expression levels of *collagen type II α 1* and *aggrecan*, while 3% L-PRF exudate only downregulated *aggrecan* expression levels. (**C**) *TGF-β* mRNA levels were not significantly altered by L-PRF ex, L-PRF CM and DPSC CM. (**D**) *MMP-13* was significantly upregulated in iMACs cultured with 25% L-PRF CM compared to the control. (**E**) *TIMP-1* mRNA expression levels were significantly upregulated by the supplementation of 25% L-PRF CM and DPSC CM. Data correspond to n = 6 for L-PRF ex and L-PRF CM and n = 7 for DPSC CM. Data are represented as mean ± S.E.M. *. $p \leq 0.05$. **. $p \leq 0.01$. ***. $p \leq 0.001$. ****. $p \leq 0.0001$.
